# Anti-Lymphangiogenic Terpenoids from the Heartwood of Taiwan Juniper, *Juniperus chinensis* var. *tsukusiensis*

**DOI:** 10.3390/plants12223828

**Published:** 2023-11-11

**Authors:** Ho-Cheng Wu, Lung-Lin Shiu, Shih-Wei Wang, Chia-Ying Huang, Tzong-Huei Lee, Ping-Jyun Sung, Yueh-Hsiung Kuo

**Affiliations:** 1 Graduate Institute of Pharmacognosy, College of Pharmacy, Taipei Medical University, Taipei 110, Taiwan; duncanwu762001@gmail.com; 2 Ph.D. Program in Clinical Drug Development of Herbal Medicine, College of Pharmacy, Taipei Medical University, Taipei 110, Taiwan; 3 School of Pharmacy, College of Pharmacy, Kaohsiung Medical University, Kaohsiung 807, Taiwan; 4 Traditional Herbal Medicine Research Center, Taipei Medical University Hospital, Taipei 110, Taiwan; 5 Department of Chemistry, National Taiwan University, Taipei 106, Taiwan; lunglin_hsu@email.eternal-group.com; 6 Institute of Biomedical Sciences, MacKay Medical College, New Taipei City 252, Taiwan; shihwei@mmc.edu.tw (S.-W.W.); u101030042@gmail.com (C.-Y.H.); 7 Department of Medicine, MacKay Medical College, New Taipei City 252, Taiwan; 8 Graduate Institute of Natural Products, College of Pharmacy, Kaohsiung Medical University, Kaohsiung 807, Taiwan; 9 Department of Chinese Medicine, MacKay Memorial Hospital, Taipei 104, Taiwan; 10 Institute of Fisheries Science, National Taiwan University, Taipei 106, Taiwan; thlee1@ntu.edu.tw; 11 National Museum of Marine Biology and Aquarium, Pingtung 944, Taiwan; pjsung@nmmba.gov.tw; 12 Department of Marine Biotechnology and Resources, National Sun Yat-sen University, Kaohsiung 804, Taiwan; 13 Department of Chinese Pharmaceutical Sciences and Chinese Medicine Resources, China Medical University, Taichung 404, Taiwan; 14 Chinese Medicine Research Center, China Medical University, Taichung 404, Taiwan; 15 Department of Biotechnology, Asia University, Taichung 413, Taiwan

**Keywords:** anti-lymphangiogenic activity, diterpenes, *Juniperus chinensis* var. *tsukusiensis*, sesquiterpenes, Taiwan juniper

## Abstract

To look in-depth into the phytochemical and pharmacological properties of Taiwan juniper, this study investigated the chemical profiles and anti-lymphangiogenic activity of *Juniperus chinensis* var. *tsukusiensis*. In this study, four new sesquiterpenes, 12-acetoxywiddrol (**1**), cedrol-13-al (**2**), α-corocalen-15-oic acid (**3**), 1,3,5-bisaoltrien-10-hydroperoxy-11-ol (**4**), one new diterpene, 1β,2β-epoxy-9α-hydroxy-8(14),11-totaradiene-3,13-dione (**5**), and thirty-three known terpenoids were successfully isolated from the heartwood of *J. chinensis* var. *tsukusiensis*. The structures of all isolates were determined through the analysis of physical data (including appearance, UV, IR, and optical rotation) and spectroscopic data (including 1D, 2D NMR, and HRESIMS). Thirty-four compounds were evaluated for their anti-lymphangiogenic effects in human lymphatic endothelial cells (LECs). Among them, totarolone (**6**) displayed the most potent anti-lymphangiogenic activity by suppressing cell growth (IC_50_ = 6 ± 1 µM) of LECs. Moreover, 3β-hydroxytotarol (**7**), 7-oxototarol (**8**), and 1-oxo-3β-hydroxytotarol (**9**) showed moderate growth-inhibitory effects on LECs with IC_50_ values of 29 ± 1, 28 ± 1, and 45 ± 2 µM, respectively. Totarolone (**6**) also induced a significant concentration-dependent inhibition of LEC tube formation (IC_50_ = 9.3 ± 2.5 µM) without cytotoxicity. The structure–activity relationship discussion of aromatic totarane-type diterpenes against lymphangiogenesis of LECs is also included in this study. Altogether, our findings unveiled the promising potential of *J. chinensis* var. *tsukusiensis* in developing therapeutics targeting tumor lymphangiogenesis.

## 1. Introduction

Lymphangiogenesis is the process of new lymphatic vessels developing from pre-existing lymphatic vasculature. It plays a role in diverse physiological scenarios, such as maintaining homeostasis, supporting the immune system, contributing to embryonic development, and aiding in wound healing. Conversely, in pathological contexts, this process is often associated with issues like organ graft rejection, lymphedema, and even cancer spread [1]. Based on the annual statistics reported by the World Health Organization, cancer is a primary contributor to mortality and a significant impediment to raising life expectancy in every nation [2]. It is worth noting that around 90% of cancer-related deaths are caused by metastatic tumor spread. Tumor lymphangiogenesis has consequently emerged as a pivotal prognostic role for cancer patients [3]. Given this understanding, developing targeted cancer therapies with anti-lymphangiogenic activity is a promising strategy to enhance patient survival rates.

The *Juniperus* species (Cupressaceae) are coniferous plants encompassing evergreen trees and shrubs, widely distributed throughout the cold temperate regions of the Northern Hemisphere to Tropical Africa [4]. The aromatic nature of *Juniperus* is due to pine oil and resin within the plant. These compounds contribute to the unique scent and flavor associated with juniper berries. Furthermore, the high resin content makes *Juniperus* wood more resistant to decay and infestation, making it a valuable material in woodworking projects. Some characteristic phytochemicals have been isolated from *Juniperus*, including acetophenones, monoterpenes, sesquiterpenes, diterpenes, flavonoids, lignans, and phenylpropanoides, mainly terpenoids [5,6,7,8,9,10,11,12,13,14,15,16,17]. The representative terpenoids in *Juniperus* include α-pinene, camphene, β-pinene, sabinene, myrcene, limonene, imbricatolic acid, junicedral, *trans*-communic acid, and isocupressic acid [18,19]. The cytotoxic lignan compounds, podophyllotoxin and deoxypodophyllotoxin, could also be found in some *Juniperus* species [20]. One of the most famous *Juniperus* plants, *J. communis* Linn., is widely used as a diuretic, antiseptic, leucorrhea, and treating gastrointestinal problems in folk medicine [4,21]. It also presents various pharmacological potentials for anti-inflammatory [22,23,24,25], antifungal [24,25,26], hepatoprotective [18,27], neuroprotective [19], anti-diabetic and anti-hyperlipidemic [28,29], and anti-proliferative activities [30,31,32,33,34]. Due to the distinctive phytochemical composition and promising pharmacological activities exhibited by *Juniperus* plants, our interest in researching them has been sparked. Our previous studies on the *J. chinensis* var. *tsukusiensis* revealed some characteristic *Juniperus* sesquiterpenoids [35,36]. However, the biological activity of *J. chinensis* var. *tsukusiensis* has never been studied, which prompted us to further explore its bioactive ingredients.

**Figure 1:**
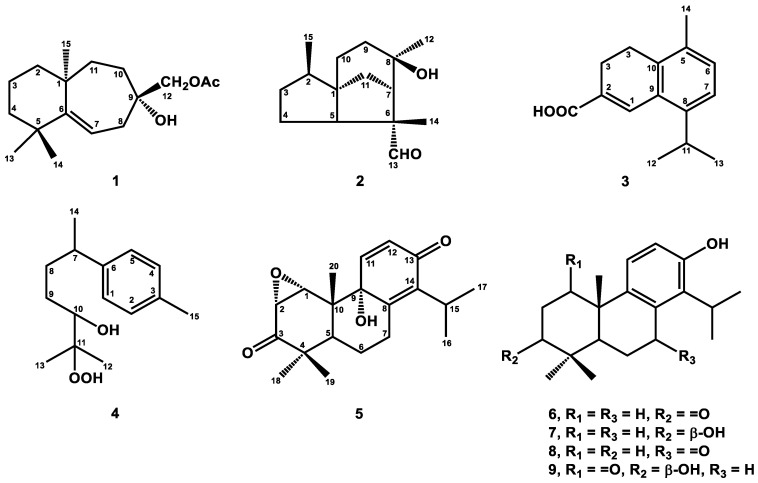
Structures of new compounds (**1**–**5**) and bioactive compounds (**6**–**9**).

In this study, a series of isolation and purification procedures were implemented, resulting in five new compounds (**1**–**5**) (Figure 1) and thirty-three known terpenoids (**6**–**38**) from the heartwood of *J. chinensis* var. *tsukusiensis*. Terpenoids are renowned for their cytotoxic effects on various types of tumors. Interestingly, in the previous investigation, it was discovered that diterpenes possess anti-lymphangiogenic properties, which is a new concept in targeting tumor cell metastasis [37,38,39]. To further explore the relationship between terpenoids and their anti-lymphangiogenic activity, as well as to expand the pharmacological profile of terpenoids, we evaluated the anti-lymphangiogenic activity of the isolated compounds in our study. Among these compounds, **6**, **7**, **8**, and **9** demonstrated significant anti-lymphangiogenic activity.

## 2. Results

### 2.1. Structure Elucidation

**Figure 2:**
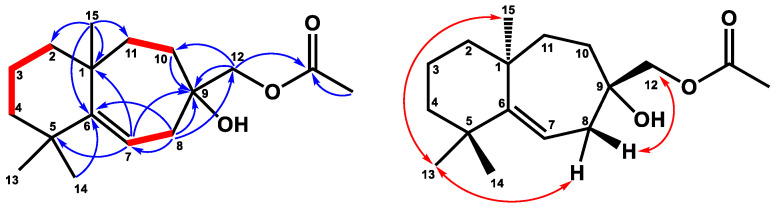
Key ^1^H–-^1^H COSY (━), HMBC (H→C), and NOESY (↔) correlations of compound **1**.

Compound **1** was obtained as a colorless, viscous oil with optical rotation. Its molecular formula was determined as C_17_H_28_O_3_ from HRESIMS data (*m/z* 281.2101 [M+H]^+^ (calcd. for 281.2117)), implying four degrees of unsaturation. The infrared spectroscopy (IR) spectrum showed typical absorptions of C=O (1742 cm^−1^) for the ester group and hydroxy group (3433 cm^−1^). The ^1^H NMR spectrum (Table 1) displayed signals of four singlet methyl groups at δ_H_ 1.04 (3H, s, H-14), 1.07 (3H, s, H-13), 1.20 (3H, s, H-15), and 2.10 (3H, s, OAc), one oxymethylene at δ_H_ 3.99 (2H, s, H-12), and one trisubstituted olefinic proton at δ_H_ 5.41 (1H, dd, *J* = 8.8, 6.0 Hz, H-7). The ^13^C NMR (Table 2) and DEPT spectra showed seventeen resonances comprising four methyls, seven methylenes, one methine, and five quaternary carbons. From the ^1^H and ^13^C NMR spectra, one C=O group (δ_C_ 171.3, C-16) and one C=C unit [δ_C_ 116.3 (C-7), 155.2 (C-6)] accounted for two of four degrees of unsaturation. Thus, compound **1** was suggested to be a bicyclic sesquiterpene with an acetyl group. The existence of the acetoxy methyl group was confirmed by the HMBC correlation (Figure 2) from H-17 to C-16 and the absorption of the ester group (1742 cm^−1^) in the IR spectrum. Comparing the ^1^H and ^13^C NMR data of **1** to those of the literature compound, widdrol [6], they shared similar structures, except for the oxidation of C-12 and an additional acetyl group in **1**. Further HMBC correlation (Figure 2) between H-12 and C-16 allowed the acetoxy group to be located at C-12. The relative stereochemistry of **1** was assigned using the information provided by the NOESY spectrum (Figure 2) and compared with the literature compound, widdrol [6]. The NOESY correlations (Figure 2) between H-13/H-8α and H-15, and H-12/H-8β, confirmed that OH-9, H-13, and H-15 were in the same phase, while H-12 and H-14 were on the same side. Thus, compound **1** was determined and named 12-acetoxywiddrol.

**Figure 3:**
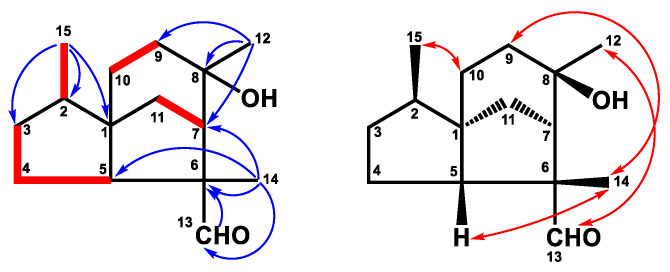
Key ^1^H–^1^H COSY (━), HMBC (H→C), and NOESY (↔) correlations of compound **2**.

Compound **2** was isolated as an optical, viscid oil, and displayed a pseudo-molecular ion at *m/z* 237.1848 [M+H]^+^ (calcd. for C_15_H_25_O_2_, 237.1855) by HRFABMS with four degrees of unsaturation. Its IR spectrum showed hydroxy and aldehyde groups at 3403 cm^−1^ and 1711 cm^−1^, respectively. The ^1^H NMR data (Table 1) depicted signals of three methyls at δ_H_ 0.84 (3H, d, *J* = 6.9 Hz, H-15), 1.31 (3H, s, H-12), and 1.41 (3H, s, H-14), and an aldehyde group with low field chemical shift at δ_H_ 9.42 (1H, s, H-1). Further ^13^C NMR (Table 2) and DEPT spectra identified three methyls, five methylenes, three methines, and four quaternary carbons, including one oxygenated quaternary carbon (δ_C_ 73.8) and one aldehyde (δ_C_ 205.6). According to the above data, the structure of **2** was assumed to be a three-membered ring sesquiterpene with an aldehyde group. A detailed comparison of the NMR data of **2** to those of 8β,13-dihydroxycedrane [40] revealed that these two compounds were structure analogous, except for the hydroxymethyl group of 8β,13-dihydroxycedrane was changed to an aldehyde group in compound **2**. The HMBC correlations (Figure 3) from H-14 to C-5 (δ_C_ 58.0), C-6 (δ_C_ 57.6), C-7 (δ_C_ 53.3), C-13 (δ_C_ 205.6), and H-13 (δ_H_ 9.42, 3H, s, H-13) to C-6 indicated that the aldehyde group was located at C-6. In the HMBC spectrum, the correlations of H-12/C-7, C-8 (δ_C_ 73.8), and C-9 (δ_C_ 34.6) verified the hydroxy group was attached at C-8. The planar structure of **2** was further supported by HMBC and COSY correlations, as shown in Figure 2. Further NOESY correlations (Figure 3) between H-15/H-10, H-14/H-5, H-9, and H-12/H-13 supported the relative configuration of **2** was the same as 8β,13-dihydroxycedrane [40]. Based on the above data, the structure of compound **2** was determined and named cedrol-13-al.

**Figure 4:**
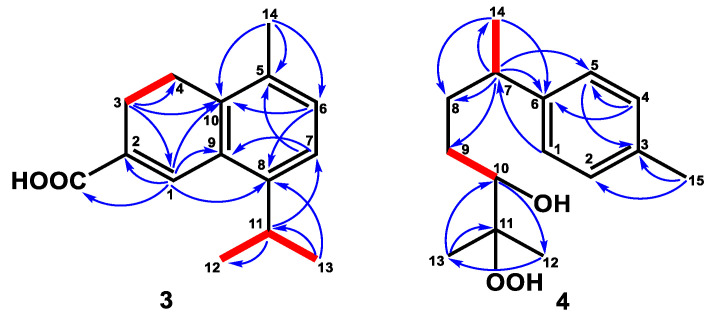
Key ^1^H–^1^H COSY (━) and HMBC (H→C) correlations of compounds **3** and **4**.

Compound **3** was yielded as a whitish solid. Analysis by HREIMS of **3** indicated a molecular formula of C_15_H_18_O_2_, representing seven IHDs. The UV spectrum showed maximum absorptions at 213, 231, and 295 nm. The IR absorption bands at 1622, 1600, and 1485 cm^−1^, as well as the observation of featuring carbon resonances (Table 2), confirmed the existence of an aromatic ring [δ_C_ 122.9 (C-6), 129.1 (C-9), 132.0 (C-7), 132.6 (C-5), 136.0 (C-10), 144.6 (C-8)] and a double bond [δ_C_ 127.7 (C-2), 135.3 (C-1)]. The carboxylic acid group was observed both in IR (1682 cm^−1^) and ^13^C NMR [δ_C_ 172.3 (C-15)] spectra. The IR spectrum also revealed the double bond (1622 cm^−1^) and aromatic ring (1600 and 1485 cm^−1^). The ^1^H NMR spectrum (Table 1) of **3** exhibited one methyl group bearing with an aromatic ring at δ_H_ 2.26 (3H, s, H-14), an isopropyl group at δ_H_ 1.23 (6H, d, *J* = 6.8 Hz, H-12 and H-13), 3.33 (1H, sept, *J* = 6.8 Hz, H-11), an *ortho*-coupling aromatic ring at δ_H_ 7.07 (1H, d, *J* = 8.0 Hz, H-6), 7.12 (1H, d, *J* = 8.0 Hz, H-7), a trisubstituted olefinic proton at δ_H_ 8.03 (1H, s, H-1), and two methylene groups at δ_H_ 2.55 (2H, t, *J* = 8.0 Hz, H-3), 2.79 (2H, t, *J* = 8.0 Hz, H-4). The HMBC experiment (Figure 4) for **3** revealed correlations between H-1/C-2, C-8, C-9, and C-10, and H-3/C-1, C-4, and C-10, confirming the 3,4-dihydronaphthalene skeleton of **3**. The HMBC correlations between H-14/C-5, C-6, and C-10 verified the methyl group (C-14) was attached to C-5 (Figure 4). The isopropyl group was located at C-8 based on the HMBC correlations between H-11/C-7 and H-13/C-8 (Figure 4). Finally, the carboxylic acid group gave cross-peak to a correlation with H-1, suggesting the carboxylic acid group was bearing with C-2. Therefore, the structure of **3** was defined as shown and named α-corocalen-15-oic acid.

Compound **4** was purified as a colorless, viscous oil. Its molecular formula of C_15_H_24_O_3_ was determined by HRESIMS (*m/z* 253.1802, calcd. for 253.1804), indicating four degrees of unsaturation. Its IR spectrum depicted a hydroxy group at 3410 cm^−1^. The ^13^C NMR (Table 2) and DEPT spectra identified four methyls [δ_C_ 20.9 (C-15), 22.4 (C-14), 23.1 (C-12), 26.5 (C-13)], an aromatic ring [δ_C_ 126.8 (C-5), 129.1 (C-4), 135.3 (C-3), 144.4 (C-6)], one oxymethine [δ_C_ 78.8 (C-10)], one oxygenated quaternary carbon [δ_C_ 73.1 (C-11)], two methylenes [δ_C_ 29.9 (C-9), 35.5 (C-8)], and one methine [δ_C_ 39.7 (C-7)]. Further ^1^H NMR spectrum (Table 1) confirmed the substituted pattern of the *para*-substituted aromatic ring [δ_H_ 7.05 (2H, d, *J* = 7.8 Hz, H-1 and H-5), 7.06 (2H, d, *J* = 7.8 Hz, H-2 and H-4)] attaching with a low field methyl group [δ_H_ 2.29 (3H, s, H-15)]. The COSY correlation (Figure 4) between H-7/H-14 and H-9/H-10 revealed the existence of fragments C-7–C-14 and C-9–C-10, respectively. The cross-peak between H-14 (δ_H_ 1.21, d, *J* = 7.2 Hz)/C-7, C-8 (δ_C_ 35.5), H-7 (δ_H_ 2.63, sex, *J* = 7.2 Hz)/C-8, C-9, H-8β (δ_H_ 1.58, m)/C-9, C-10, and H-13 (δ_H_ 1.11, s)/C-10, C-11, and C-12 in the HMBC spectrum (Figure 4) suggested the existence of the side-chain group. Further analysis of the HMBC correlations (Figure 4) between H-7/C-1, C-5, and C-6 showed that the location of the side-chain group was connected with the aromatic ring at C-6. According to the carbon and HRESIMS spectra, compound **4** contained two oxygenated carbons (C-10 and C-11) with three oxygen atoms, implying the existence of a hydroxy and a hydroperoxy group. Based on the chemical shift of H-10 (δ_H_ 3.26), the hydroxy group was attached to C-10, and the hydroperoxy group was connected with C-11, respectively. The structure of **4** was accordingly confirmed and named 1,3,5-bisaoltrien-10-hydroperoxy-11-ol.

**Figure 5:**
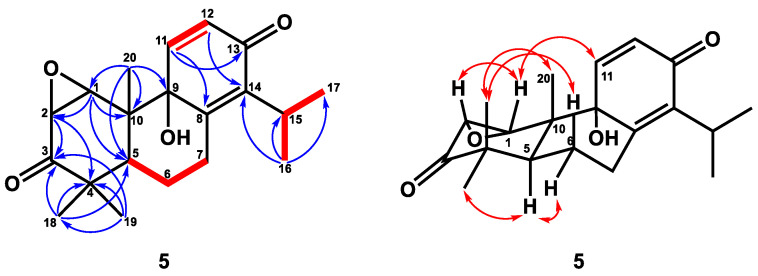
Key ^1^H–^1^H COSY (━), HMBC (H→C), and NOESY (↔) correlations of compound **5**.

Compound **5** was obtained as a colorless crystal with optical rotation. A molecular formula of C_20_H_26_O_4_ was determined for this compound by HRESIMS (*m/z* 331.1906, calcd. for 331.1909), with eight degrees of unsaturation. The IR spectrum of **5** showed IR absorption bands at 3417 cm^−1^ for a hydroxy group, at 1699 cm^−1^ for one carbonyl group [δ_C_ 211.0 (C-3)], and at 1669 cm^−1^ for one conjugated carbonyl group [δ_C_ 184.6 (C-13)], which was supported by analysis of the ^13^C NMR spectrum (Table 2). The IR spectrum also revealed the presence of C=C double bonds (1631, 990, 840 cm^−1^). The ^1^H NMR spectrum of **5** exhibited three singlet methyl groups [δ_H_ 0.71 (3H, s, H-20), 0.98 (3H, s, H-19), and 1.20 (3H, s, H-18)], an isopropyl group [δ_H_ 1.21 (3H, d, *J* = 7.2 Hz, H-17), 1.23 (3H, d, *J* = 7.2 Hz, H-16)], and 3.22 (1H, sept, *J* = 7.2 Hz, H-15)], two oxymethines [δ_H_ 3.38 (1H, d, *J* = 4.6 Hz, H-2), 3.64 (1H, d, *J* = 4.6 Hz, H-1)], and double-bond signals [δ_H_ 6.28 (1H, d, *J* = 10.0 Hz, H-12), 7.01 (1H, d, *J* = 10.0 Hz, H-11)]. The ^13^C NMR and DEPT spectra indicated the presence of five methyls [δ_C_ 13.9 (C-20), 20.8 (C-19), 21.4 (C-17), 21.5 (C-16), 28.4 (C-18)], two oxymethines with epoxy ring [δ_C_ 54.5 (C-2), 60.9 (C-1)], one oxygenated quaternary carbon [δ_C_ 74.2 (C-9)], two pairs of double bonds [δ_C_ 131.3 (C-12)/144.9 (C-11), 144.0 (C-14)/153.7 (C-8)], two carbonyl groups [δ_C_ 184.6 (C-13), 211.0 (C-3)], two quaternary carbon [δ_C_ 44.8 (C-4), 46.7 (C-10)], two methines [δ_C_ 26.6 (C-15), 38.6 (C-55)], and two methylenes [δ_C_ 24.0 (C-6), 26.6 (C-7)]. From the above spectroscopic data, **5** was assumed to be a totarane diterpene similar to totarolone [41], except for an additional epoxy group at C-1/C-2, a hydroxy group at C-9, and the aromatic ring in totarolone was changed to a cyclohexa-2,5-dien-1-one ring in **5**. The HMBC correlations (Figure 5) between H-20/C-1; H-1/C-2, C-5, and C-10; and H-2/C-3, C-4 indicated C-1 and C-2 were connected with epoxy group. Moreover, the *cis* disposition of H-1/H-2 was confirmed by the small coupling constant of H-1/H-2 (*J* = 4.6 Hz). The hydroxy group was located at C-9 from HMBC correlations of H20/C-9 and H-12/C-9. The existence of the cyclohexa-2,5-dien-1-one unit was verified based on the HMBC correlations between H-11/C-8, C-13 and H-12/C-9, C-14. The NOESY correlation (Figure 5) between H-19/H-6β, H-20 and H-5/H-6α, H-18, indicating H-19 and H-20 were in axial position. The NOESY correlations of H-1/H-2 and H-11 suggested that H-1, H-2, and H-11 were in the same phase (Figure 5). Additionally, the epoxy group and OH-9 were at the α-position based on the ring junction afforded by NOESY correlations. Thus, the structure of compound **5** was elucidated as 1β,2β-epoxy-9α-hydroxy-8(14),11-totaradiene-3,13-dione.

By comparing the spectroscopic data ([a]_D_, UV, IR, NMR, and MS) of known compounds with the literature data, the known diterpenes were identified to be totarolone (**6**) [41], 3β-hydroxytotarol (**7**) [42], 7-oxototarol (**8**) [41], and 1-oxo-3β-hydroxytotarol (**9**) [41], 5,6-dehydrosugiol methyl ether (**10**) [43], sandaracopimaric acid (**11**) [35], 3,18-dihydroxypimara-8(14),15-diene (**12**) [44], secoabietane dialdehyde (**13**) [43], communic acid (**14**) [43], and (12*R*, 13*S*)-dihtdroxylabda-8(17),14-dien-19-oic acid (**15**) [45]. Additionally, the known sesquiterpenes were identified to be 2-himachalen-6-ol (**16**) [36], 3-himachalen-6-ol (**17**) [36], chinensiol (**18**) [36], *ar*-himachalene (**19**) [36], 12-hydroxy-α-longipinene (**20**) [35], 15-hydroxyacora-4(14),8-diene (**21**) [35], cedrol (**22**) [35], 8α,12-cedranediol (**23**) [46], 8-cedren-13-ol (**24**) [8], epicedrane-diol (**25**) [47], cedrol-13-oic acid (**26**) [48], 13-acetoxycedrol (**27**) [48], widdrol (**28**) [35], (+)-α-bisabolol (**29**) [7], sesquithuriferol (**30**) [8], 15-hydroxyallo-cedrol (**31**) [7], thujopsenal (**32**) [35], hinokiic acid (**33**) [35], mayurone (**34**) [49], 4α,5α-epoxymayurone (**35**) [50], 8β-hydroxythujopsan-9-one (**36**) [51], nootkatone (**37**) [52], and γ-cuparanol (**38**) [53].

### 2.2. Anti-Lymphangiogenic Effects of Compounds Isolated from J. chinensis var. tsukusiensis

**Figure 6:**
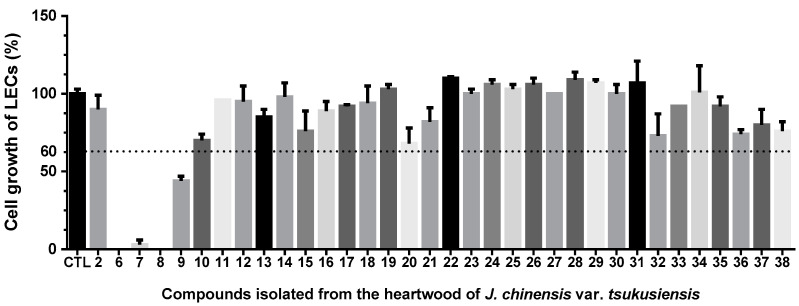
Anti-lymphangiogenic effects of compounds in human LECs. LECs were treated with the indicated compounds at a concentration of 50 μM for 48 h, and anti-lymphangiogenic effects were elucidated in a cell growth assay (*n* = 3). Data were expressed as the mean ± SEM.

The role of lymphangiogenesis in tumor development, progression, and metastasis has been well-documented in various human cancers. Therefore, our study focused on examining the anti-lymphangiogenic effects of 34 compounds in human lymphatic endothelial cells (LECs). As shown in Figure 6, compounds **6**–**9** showed growth-inhibitory effects on LECs (the percentage of LECs growth < 60%). These active isolates were further evaluated for their IC_50_ values of anti-lymphangiogenic activity (Table 3). As shown in Table 3, totarolone (**6**) exhibited the most potent anti-lymphangiogenic activity by suppressing LEC growth (IC_50_ = 6 ± 1 µM), with rapamycin as the positive control. 3β-Hydroxytotarol (**7**), 7-oxototarol (**8**), and 1-oxo-3β-hydroxytotarol (**9**) showed moderate growth-inhibitory effects on LECs with IC_50_ values of 29 ± 1, 28 ± 1, and 45± 2 µM, respectively.

**Figure 7:**
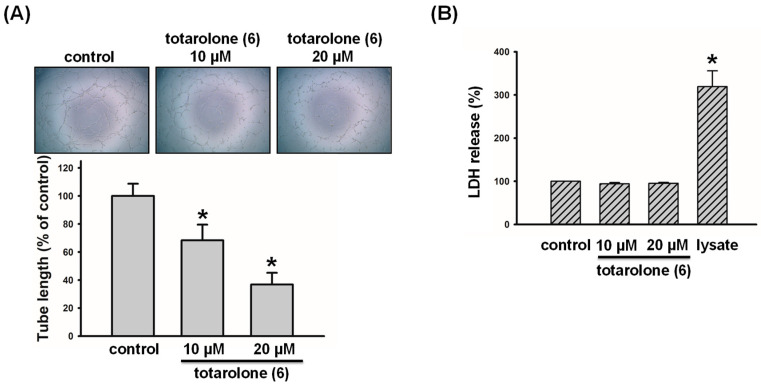
Effects of compound **6** on tube formation and cytotoxicity in human LECs. (**A**) Cells were treated with compound **6** (10 and 20 μM) for 8 h, and tubular morphogenesis was recorded by an inverted phase-contrast microscope (*n* = 3). ImageJ 1.54a software was used to quantify the length of capillary-like tubes. (**B**) LECs were treated with compound **6** (10 and 20 μM), then the cytotoxicity was evaluated by the LDH assay (*n* = 3). Data are expressed as the mean ± SEM. * *p* < 0.05 compared with the control group.

To confirm the anti-lymphangiogenic effects of the active compounds, we proceeded with the capillary tube formation assay. As illustrated in Figure 7, compound **6** induced the promising anti-lymphangiogenesis property by disrupting LECs tube formation in a concentration-dependent manner (IC_50_ = 9.3 ± 2.5 µM). Furthermore, it was observed that compound **6** did not increase the levels of lactate dehydrogenase (LDH) in LECs. The results indicate that compound **6** exerts significant anti-lymphangiogenic effects without cytotoxic fashion.

## 3. Discussion

In this study, compounds isolated from the heartwood of *J. chinensis* var. *tsukusiensis* can be categorized into sesquiterpenoids and diterpenoids. Among the tested compounds, only aromatic totarane-type diterpenes depicted anti-lymphangiogenic effects in human LECs. An in-depth discussion of the structure–activity relationship (SAR) in tested compounds was raised. Totarolone (**6**) showed better anti-lymphangiogenic activity than 7-oxototarol (**8**), suggesting that the substituted position of the carbonyl group is crucial for anti-lymphangiogenic activity. Surprisingly, 3β-hydroxytotarol (**7**), a compound reduction by the 3-carbonyl group in totarolone (**6**), exhibits lower anti-lymphangiogenic activity than totarolone (**6**). Moreover, 3β-hydroxytotarol (**7**) provided better anti-lymphangiogenic activity than 1-oxo-3β-hydroxytotarol (**9**), implying that 3-carbonyl substituent on aromatic totarane-type diterpene may attenuate the anti-lymphangiogenic activity.

## 4. Material and Methods

### 4.1. General Experiment Procedures

Optical rotations were measured on a Jasco DIP-1000 Digital polarimeter (Jasco, Kyoto, Japan), and IR spectra (neat) were acquired with a Perkin-Elmer 983G spectrometer (Perkin-Elmer, Waltham, MA, USA). The 1D (^1^H, ^13^C, DEPT) and 2D (COSY, NOESY, HSQC, HMBC) NMR spectra were obtained from a Burcher DMX-300 spectrometer (Bruker Inc., Bremen, Germany) operated at 300 (^1^H) and 75 MHz (^13^C), Varian Unityplus-400 spectrometer (Varian, Inc. Vacuum Technologies, Lexington, MA, USA) operated at 400 (^1^H) and 100 MHz (^13^C). The HRESIMS data were generated at the Mass Spectrometry Laboratory by Orbitrap QE Plus Mass Spectrometry (Thermo Fisher Scientific, Inc., Bremen, Germany). Extracts were chromatographed on silica gel (70–230 mesh, 230–400 mesh, Merk, Darmstadt, Germany, ASTM) and purified with a semipreparative normal-phase HPLC column (Merck LichroCART 250 mm × 10 mm, 7 µm, LiChrosorb Si 60, Merck, Darmstadt, Germany) taken on LDC Analytical-III.

### 4.2. Plant Material

The heartwood of *Juniperus chinensis* Linn. var. *tsukusiensis* Masam. was collected in Chingshui Mountain, Hualien, Taiwan, in October 1990 and identified by Dr. Sheng-yYou Lu at the Taiwan Forestry Research Institute.

### 4.3. Extraction and Isolation

The dried heartwood of *J. chinensis* var. *tsukusiensis* (4 kg) was extracted at room temperature with MeOH (80 L) four times (4 days for each time) to yield an MeOH extract that was partitioned between *n*-hexane and H_2_O (1:1) to provide an *n*-hexane-soluble (230 g) and an H_2_O-soluble layer. The *n*-hexane-soluble layer was subjected to column chromatography (silica gel; 2.3 kg; started from 100% *n*-hexane, then washed with EtOAc in gradient mode, and finally washed with 100% EtOAc, 100% acetone, and 100% methanol, respectively) to yield 15 subfractions (Fr. A–~Fr. O). Fr. J was subjected to an HPLC column (silica gel; *n*-hexane/dichloromethane/EtOAc = 75/5/20) to obtain compound **2** (21.3 mg). Fr. L was separated with an HPLC column (silica gel; *n*-hexane/dichloromethane/EtOAc = 60/20/20) to gain compounds **4** (3.4 mg) and **5** (4.0 mg). Fr. M was purified on an HPLC column (silica gel; *n*-hexane/dichloromethane/EtOAc = 40/20/40) to furnish compound **1** (11.5 mg). Fr. N was re-chromatographed on an HPLC column (silica gel; *n*-hexane/dichloromethane/EtOAc = 20/20/60) to afford compound **3** (3.2 mg). The purification process of known compounds can be retrieved from the Supplementary Information (Table S1).

### 4.4. Spectroscopic Data of Compounds

#### 4.4.1. 12-Acetoxywiddrol (**1**)

Viscid oil. [α]$\begin{array}{c} 25\\ D \end{array}$: +71.3 (*c* 1.0, CHCl_3_). IR *v*_max_: 3433 (OH), 1742 (C=O) cm^−1^. ^1^H NMR (CDCl_3_, 300 MHz): see Table 1. ^13^C NMR (CDCl_3_, 75 MHz): see Table 2.

#### 4.4.2. Cedrol-13-al (**2**)

Viscid oil. [α]$\begin{array}{c} 25\\ D \end{array}$: −35.3 (*c* 1.9, CHCl_3_). IR *v*_max_: 3403 (OH), 1711 (C=O) cm^−1^. ^1^H NMR (CDCl_3_, 300 MHz): see Table 1. ^13^C NMR (CDC1_3_, 75 MHz): see Table 2. HRESIMS *m/z*: 237.1848 [M+H]^+^ (calcd. for C_15_H_25_O_2_, 237.1855).

#### 4.4.3. α-Corocalen-15-oic Acid (**3**)

Whitish solid. IR *v*_max_: 3300–2500 (OH), 1682 (C=O), 1622 (C=C), 1600, 1485 (aromatic ring) cm^−1^. ^1^H NMR (CDCl_3_, 400 MHz): see Table 1. ^13^C NMR (CDC1_3_, 100 MHz): see Table 2. HRESIMS *m/z*: 231.1831 [M+H]^+^ (calcd. for C_15_H_19_O_2_, 231.1835).

#### 4.4.4. 1,3,5-Bisaoltrien-10-hydroperoxy-11-ol (**4**)

Viscid oil. [α]$\begin{array}{c} 25\\ D \end{array}$: +6.0 (*c* 0.3, CHCl_3_). IR *v*_max_: 3410 (OH), 1514 (aromatic ring) cm^−1^. ^1^H NMR (CDCl_3_, 400 MHz): see Table 1. ^13^C NMR (CDCl_3_, 100 MHz): see Table 2. HRESIMS *m/z*: 253.1802 [M+H]^+^ (calcd. for C_15_H_25_O_3_, 253.1804).

#### 4.4.5. 1β,2β-Epoxy-9α-hydroxy-8(14),11-totaradiene-3,13-dione (**5**)

Colorless crystal. [α]$\begin{array}{c} 25\\ D \end{array}$: +79.4 (*c* 0.4, CHCl_3_). IR *v*_max_: 3417 (OH), 1699, 1669 (C=O), 1631 (C=C) cm^−1^. ^1^H NMR (CDCl_3_, 400 MHz): see Table 1. ^13^C NMR (CDC1_3_, 75 MHz): see Table 2. HRESIMS *m/z*: 331.1906 [M+H]^+^ (calcd. for C_20_H_27_O_4_, 331.1909).

### 4.5. Anti-Lymphangiogenic Assay

The methods employed for cell culture, cell growth, tube formation, and cytotoxicity assessments of human lymphatic endothelial cells were consistent with our previous work [54].

## 5. Conclusions

Phytochemical investigation of the heartwood of *J. chinensis* var. *tsukusiensis* led to 38 compounds, including 5 new compounds and 33 known compounds in this study. The chemical components of *Juniperus* species have been well-studied; however, more studies focus on *J. communis* rather than *J. chinensis* var. *tsukusiensis*. The phytochemical findings in this study not only contribute to identifying additional natural sources of terpenoids but also enhance our understanding of the chemical profiles of *J. chinensis* var. *tsukusiensis*. Additionally, 34 isolates from *J. chinensis* var. *tsukusiensis* screened their anti-lymphangiogenic effects in human lymphatic endothelial cells. It is noticeable that this is the first report on the anti-lymphangiogenic activities of *J. chinensis* var. *tsukusiensis* and demonstrates the potential of totarolone (**6**), 3β-hydroxytotarol (**7**), 7-oxototarol (**8**), and 1-oxo-3β-hydroxytotarol (**9**) to develop therapeutics against tumor lymphangiogenesis.

## Figures and Tables

**Table 1:** ^1^H NMR data of compounds **1**–**5** in CDCl_3_.

Position	1 ^a^	2 ^a^	3 ^b^	4 ^b^	5 ^b^
	*δ*_H_ (mult, *J* in Hz)	*δ*_H_, mult (*J* in Hz)	*δ*_H_, mult (*J* in Hz)	*δ*_H_, mult (*J* in Hz)	*δ*_H_, mult (*J* in Hz)
1			8.03 (s)	7.05 (d, 7.8)	3.64 (d, 4.6)
2	1.23 (m) 1.49 (m)	1.62 (m)		7.06 (d, 7.8)	3.38 (d, 4.6)
3	1.48 (m) 1.72 (m)	1.23 (m) 1.88 (m)	2.55 (t, 8.0)		
4	1.32 (m) 1.79 (m)	1.24 (m) 1.80 (m)	2.79 (t, 8.0)	7.06 (d, 7.8)	
5		1.9 (t, 7.6) (β)		7.05 (d, 7.8)	
6			7.07 (d, 8.0)		1.38 (dd, 13.3, 4.0) (β) 1.81 (m) (α)
7	5.41 (dd, 8.8, 6.0)	2.26 (d, 4.4)	7.12 (d, 8.0)	2.63 (sex, 7.2)	2.58 (m) (β) 2.94 (m) (α)
8	2.06 (dd, 14.0, 8.8) (β) 2.46 (dd, 14.0, 6.0) (α)			1.58 (m) 1.84 (m)	
9		1.71 (dt, 5.6, 1.6) (β) 1.82 (m) (α)		1.28 (m) 1.32 (m)	
10	1.33 (m) 1.62 (m)	1.35 (m) (β) 1.44 (m) (α)		3.26 (dd, 10.0, 2.4)	
11	1.24 (m) 1.41 (m)	1.42 (m) 1.76 (m)	3.33 (sept, 6.8)		7.01 (d, 10.0)
12	3.99 (s)	1.31 (s)	1.23 (d, 6.8)	1.08 (s)	6.28 (d, 10.0)
13	1.07 (s)	9.42 (s)	1.23 (d, 6.8)	1.11 (s)	
14	1.04 (s)	1.41 (s)	2.26 (s)	1.21 (d, 7.2)	
15	1.20 (s)	0.84 (d, 7.2)		2.29 (s)	3.22 (sept, 7.2)
16					1.23 (d, 7.2)
17	2.10 (s)				1.21 (d, 7.2)
18					1.20 (s)
19					0.98 (s)
20					0.71 (s)

^a^ Data measured at 300 MHz. ^b^ Data measured at 400 MHz.

**Table 2:** ^13^C NMR data of compounds **1**–**5** in CDCl_3_.

Position	1 ^a^	2 ^a^	3 ^b^	4 ^b^	5 ^b^
	*δ*_C_	*δ*_C_	*δ*_C_	*δ*_C_	*δ*_C_
1	39.8	53.7	135.3	126.8	60.9
2	41.4	41.0	127.7	129.1	54.5
3	18.6	37.8	21.0	135.3	211.0
4	38.1	25.9	24.6	129.1	44.8
5	36.9	58.0	132.6	126.8	38.6
6	155.2	57.6	122.9	144.4	24.0
7	116.3	53.3	132.0	39.7	26.6
8	34.4	73.8	144.6	35.5	153.7
9	73.8	34.6	129.1	29.9	74.2
10	32.7	42.6	136.0	78.8	46.7
11	40.5	30.8	28.5	73.1	144.9
12	70.2	30.2	23.8	23.1	131.3
13	31.7	205.6	23.8	26.5	184.6
14	32.9	19.3	19.4	22.4	140.0
15	27.5	15.4	172.3	20.9	26.6
16	171.3				21.5
17	20.9				21.4
18					28.4
19					20.8
20					13.9

^a^ Data measured at 75 MHz. ^b^ Data measured at 100 MHz.

**Table 3:** Anti-lymphangiogenic activity of selected compounds in human LECs.

Compound	IC_50_ (µM)
totarolone (**6**)	6 ± 1
3β-hydroxytotarol (**7**)	29 ± 1
7-oxototarol (**8**)	28 ± 1
1-oxo-3β-hydroxytotarol (**9**)	45 ± 2
Rapamycin ^#^	<10

LECs were treated with compounds **6**–**9** for 48 h, and anti-lymphangiogenic effects were elucidated in a cell growth assay (*n* = 3). Data were expressed as the mean ± SEM. ^#^ Rapamycin was used as a positive control.

## Data Availability

Data are contained within the article and supplementary materials.

## Supplementary Materials

The following supporting information can be downloaded at https://www.mdpi.com/article/10.3390/plants12223828/s1, including Table S1: Isolation and purification methods of known compounds isolated from *J. chinensis* var. *tsukusiensis*; Figure S1–S15: The phytochemical spectra of compounds **1**–**5**.
