# Supplementary material for: Anti-Lymphangiogenic Terpenoids from the Heartwood of Taiwan Juniper, Juniperus chinensis var. tsukusiensis
Source: Plants (Basel). 2023 Nov 11;12(22):3828. doi: 10.3390/plants12223828 (PMC10674874; doi:10.3390/plants12223828)
Supplement: Supplementary file 1 [file plants-12-03828-s001.zip › plants-2708352-supplementary.pdf]

Anti-Lymphangiogenic Terpenoids from the Heartwood of Taiwan Juniper, *Juniperus chinensis* var. *tsukusiensis*

Ho-Cheng Wu [1,2,3,4], Lung-Lin Shiu [5], Shih-Wei Wang [6,7,8], Chia-Ying Huang [6,9], Tzong-Huei Lee [10], Ping-Jyun Sung [11,12] and Yueh-Hsiung Kuo [13,14,15,*]

1 Graduate Institute of Pharmacognosy, College of Pharmacy, Taipei Medical University, Taipei 110, Taiwan; duncanwu762001@gmail.com
2 Ph.D. Program in Clinical Drug Development of Herbal Medicine, College of Pharmacy, Taipei Medical University, Taipei 110, Taiwan
3 School of Pharmacy, College of Pharmacy, Kaohsiung Medical University, Kaohsiung 807, Taiwan
4 Traditional Herbal Medicine Research Center, Taipei Medical University Hospital, Taipei 110, Taiwan
5 Department of Chemistry, National Taiwan University, Taipei 106, Taiwan; lunglin_hsu@email.eternal-group.com
6 Institute of Biomedical Sciences, MacKay Medical College, New Taipei City 252, Taiwan; shihwei@mmc.edu.tw (S.-W.W.); u101030042@gmail.com (C.-Y.H.)
7 Department of Medicine, MacKay Medical College, New Taipei City 252, Taiwan
8 Graduate Institute of Natural Products, College of Pharmacy, Kaohsiung Medical University, Kaohsiung 807, Taiwan
9 Department of Chinese Medicine, MacKay Memorial Hospital, Taipei 104, Taiwan
10 Institute of Fisheries Science, National Taiwan University, Taipei 106, Taiwan; thlee1@ntu.edu.tw
11 National Museum of Marine Biology and Aquarium, Pingtung 944, Taiwan; pjsung@nmmba.gov.tw
12 Department of Marine Biotechnology and Resources, National Sun Yat-sen University, Kaohsiung 804, Taiwan
13 Department of Chinese Pharmaceutical Sciences and Chinese Medicine Resources, China Medical University, Taichung 404, Taiwan
14 Chinese Medicine Research Center, China Medical University, Taichung 404, Taiwan
15 Department of Biotechnology, Asia University, Taichung 413, Taiwan
* Correspondence: kuoyh@mail.cmu.edu.tw; Tel.: +886-4-2205-3366 (ext. 5701)

Supplementary data

Table S1. Isolation and purification methods of known compounds isolated from *J. chinensis* var. *tsukusiensis* 3
Figure S1. ^{1}H NMR spectrum of 12-acetoxywiddrol (**1**) in $CDCl_3$ at 300 MHz 4
Figure S2. DEPT spectrum of 12-acetoxywiddrol (**1**) 4
Figure S3. HRESIMS spectrum of 12-acetoxywiddrol (**1**) 5
Figure S4. ^{1}H NMR spectrum of cedrol-13-al (**2**) in $CDCl_3$ at 300 MHz 5
Figure S5. DEPT spectrum of cedrol-13-al (**2**) 6
Figure S6. HRESIMS spectrum of cedrol-13-al (**2**) 6
Figure S7. ^{1}H NMR spectrum of α-corocalen-15-oic acid (**3**) in $CDCl_3$ at 400 MHz 7
Figure S8. DEPT spectrum of α-corocalen-15-oic acid (**3**) 7
Figure S9. HRESIMS spectrum of α-corocalen-15-oic acid (**3**) 8
Figure S10. ^{1}H NMR spectrum of 1,3,5-bisaoltrien-10-hydroperoxy-11-ol (**4**) in $CDCl_3$ at 400 MHz 8
Figure S11. DEPT spectrum of 1,3,5-bisaoltrien-10-hydroperoxy-11-ol (**4**) 9
Figure S12. HRESIMS spectrum of 1,3,5-bisaoltrien-10-hydroperoxy-11-ol (**4**) 9
Figure S13. ^{1}H NMR spectrum of 1β,2β-epoxy-9α-hydroxy-8(14),11-totaradiene-3,13-dione (**5**) in $CDCl_3$ at 400 MHz 10
Figure S14. DEPT spectrum of 1β,2β-epoxy-9α-hydroxy-8(14),11-totaradiene-3,13-dione (**5**) in $CDCl_3$ at 400 MHz 10
Figure S15. HRESIMS spectrum of 1β,2β-epoxy-9α-hydroxy-8(14),11-totaradiene-3,13-dione (**5**) in $CDCl_3$ at 400 MHz 11

Table S1. Isolation and purification methods of known compounds isolated from *J. chinensis* var. *tsukusiensis*

Fraction	HPLC method	Obtained compounds (mg)
A	EtOAc:CH_2Cl_2:*n*-Hexane = 5:5:90	**34** (75.4 mg), **35** (26.4 mg), **36** (10.0 mg)
B	EtOAc:CH_2Cl_2:*n*-Hexane = 10:10:80	**8** (2.0 mg), **10** (8.6 mg)
C	EtOAc:CH_2Cl_2:*n*-Hexane = 5:5:90	**16** (5.6 mg)
D	EtOAc:CH_2Cl_2:*n*-Hexane = 10:10:80	**19** (2.3 mg), **30** (10.0 mg), **36** (10.0 mg)
E	EtOAc:CH_2Cl_2:*n*-Hexane = 10:10:80	**29** (3.5 mg)
F	EtOAc:CH_2Cl_2:*n*-Hexane = 10:5:85	**29** (3.0 mg)
G	EtOAc:CH_2Cl_2:*n*-Hexane = 10:5:85	**22** (450.2 mg)
H	EtOAc:*n*-Hexane = 20:80	**11** (142.0 mg), **14** (16.0 mg), **20** (24.3 mg), **21** (6.7 mg), **24** (5.6 mg), **28** (35.6 mg), **32** (16.7 mg), **37** (15.0 mg),
I	EtOAc:CH_2Cl_2:*n*-Hexane = 20:5:75	**6** (3.0 mg), **13** (12.0 mg), **38** (10.0 mg)
J	EtOAc:CH_2Cl_2:*n*-Hexane = 20:5:75	**26** (2.3 mg), **33** (363.0 mg)
K	EtOAc:*n*-Hexane = 30:70	**7** (12.0 mg)
L	EtOAc:CH_2Cl_2:*n*-Hexane = 20:20:60	**9** (3.6 mg), **15** (1.2 mg), **18** (3.8 mg), **23** (25.6 mg), **31** (13.7 mg),
M	EtOAc:CH_2Cl_2:*n*-Hexane = 40:20:40	**12** (6.5 mg), **17** (35.5 mg)
N	EtOAc:CH_2Cl_2:*n*-Hexane = 60:20:20	**25** (13.5 mg), **27** (3.2 mg)
O	EtOAc:CH_2Cl_2:*n*-Hexane = 60:20:20	

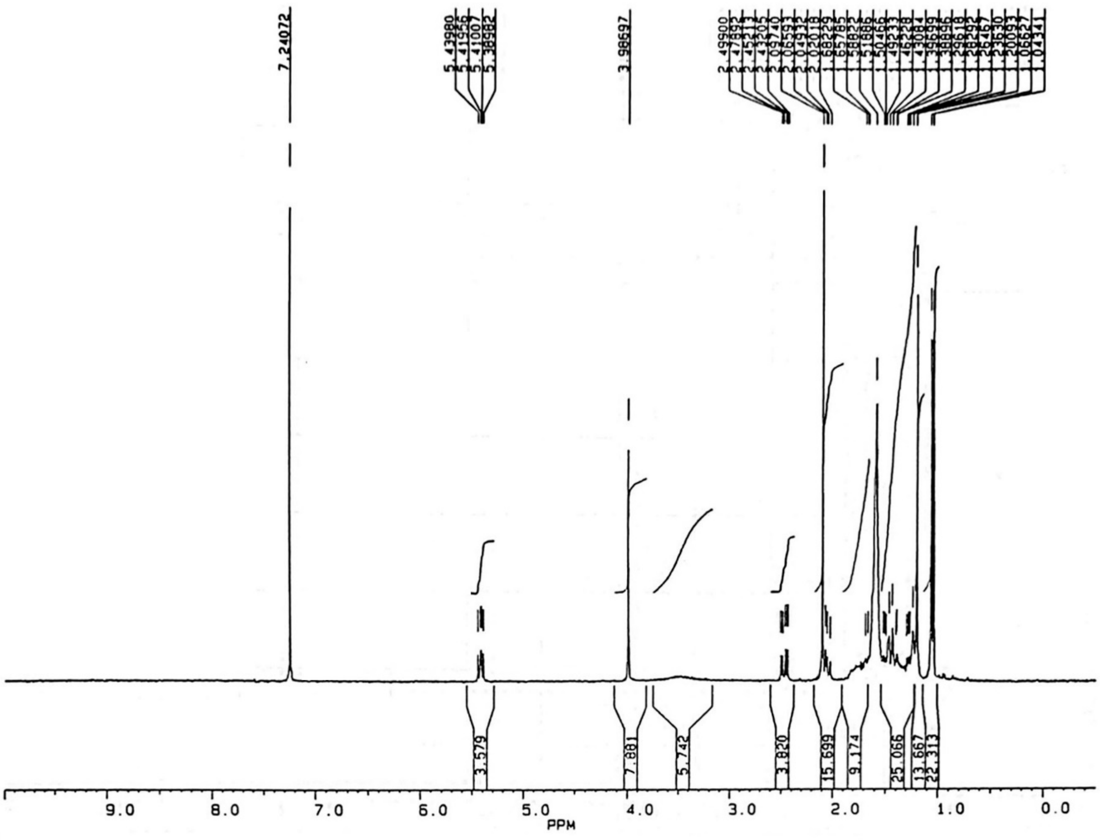

Figure S1. ^{1}H NMR spectrum of 12-acetoxywiddrol (**1**) in $CDCl_3$ at 300 MHz

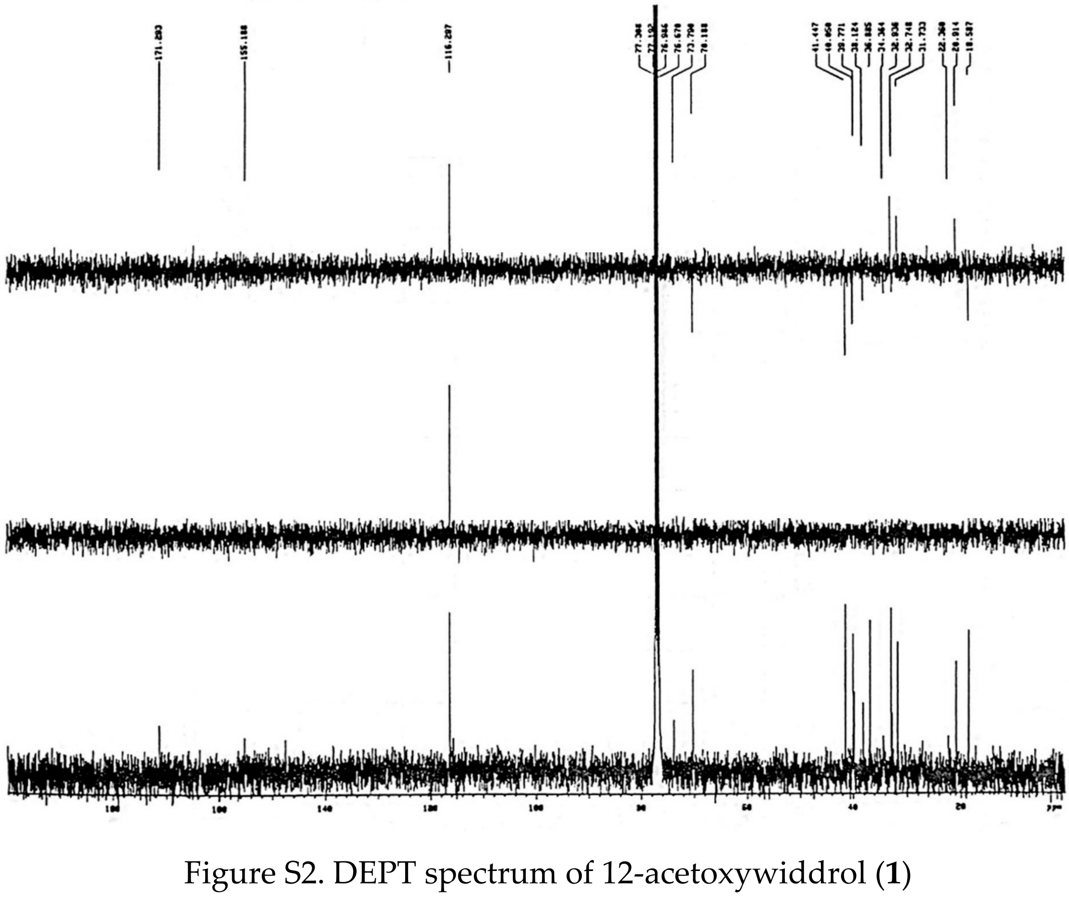

Figure S2. DEPT spectrum of 12-acetoxywiddrol (**1**)

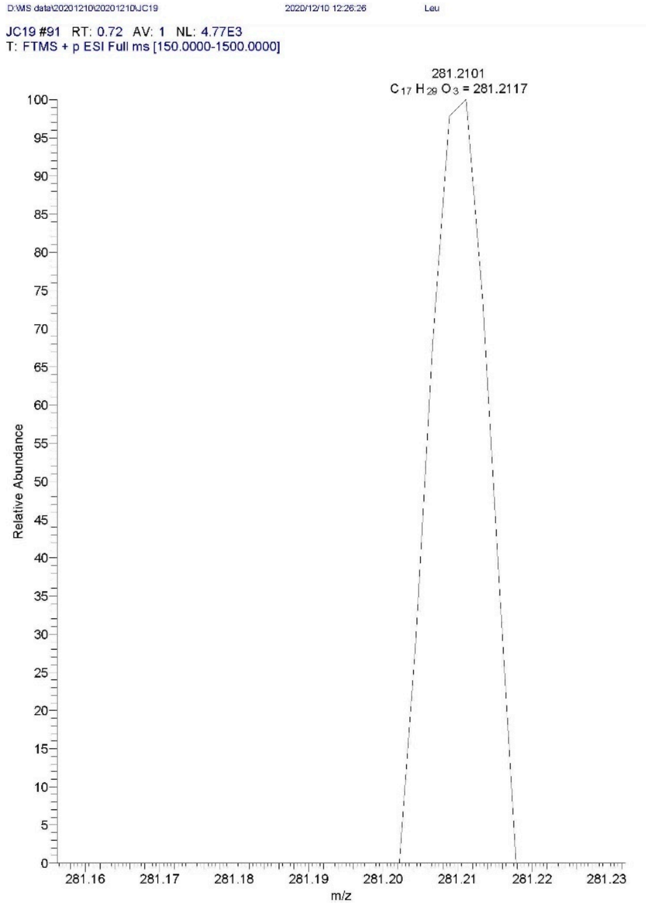

Figure S3. HRESIMS spectrum of 12-acetoxywiddrol (**1**)

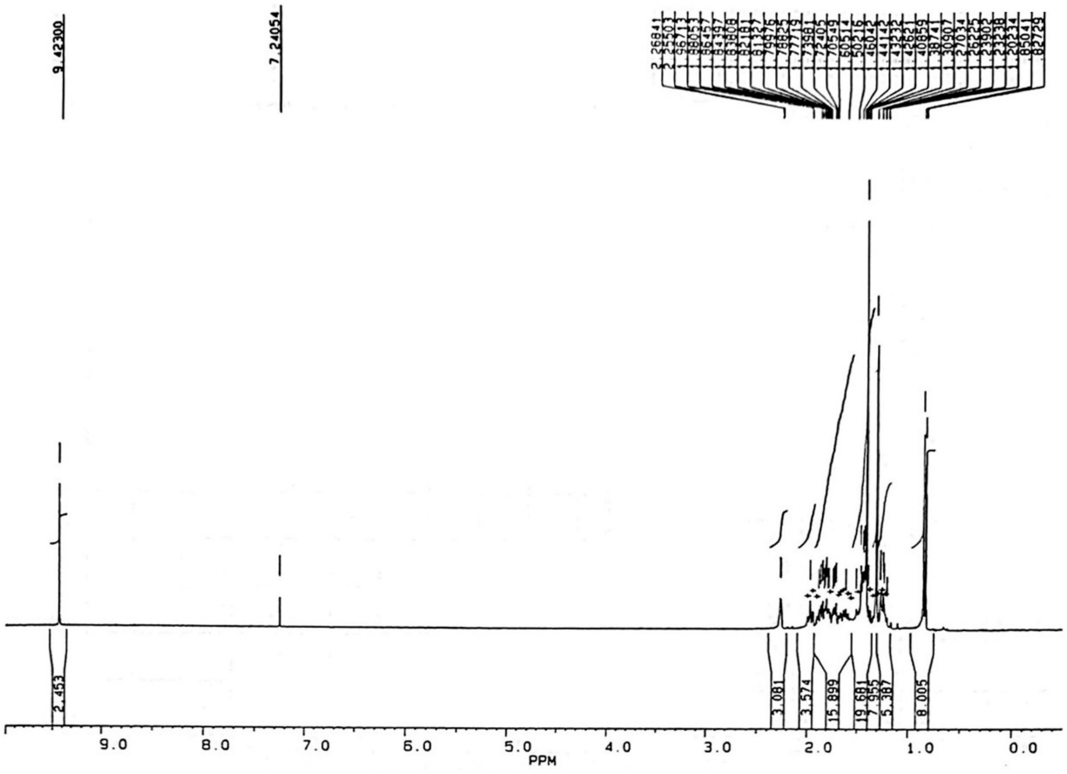

Figure S4. ^{1}H NMR spectrum of cedrol-13-al (**2**) in $CDCl_3$ at 300 MHz

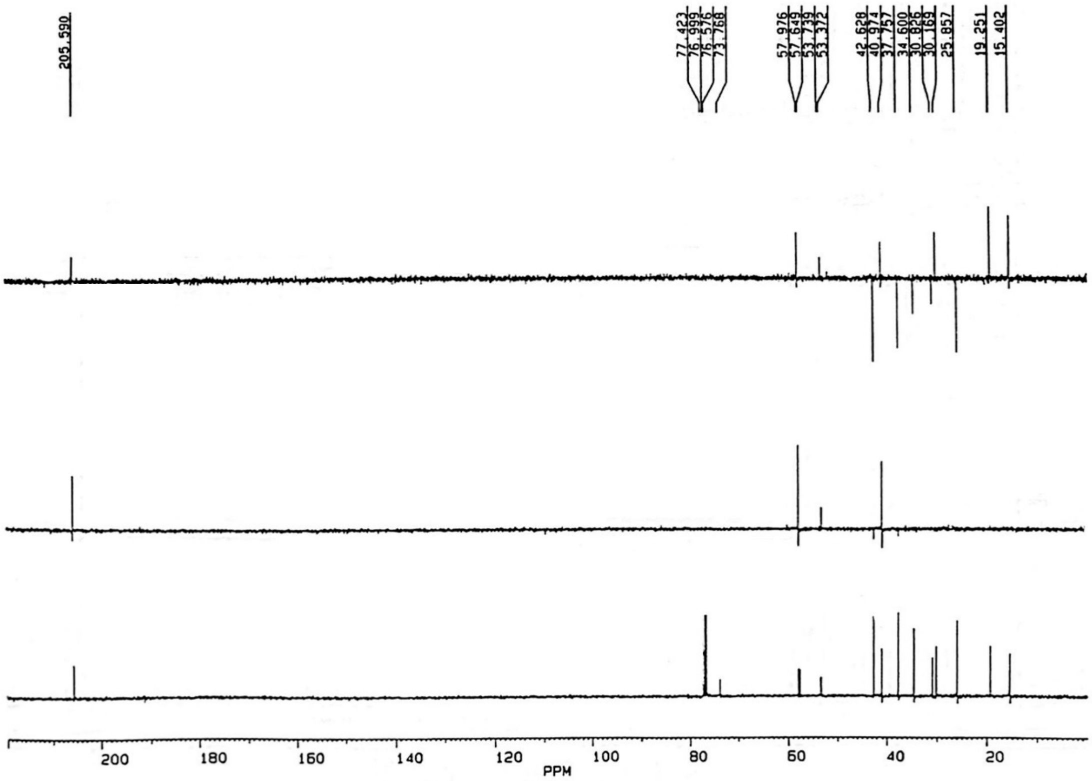

Figure S5. DEPT spectrum of cedrol-13-al (**2**)

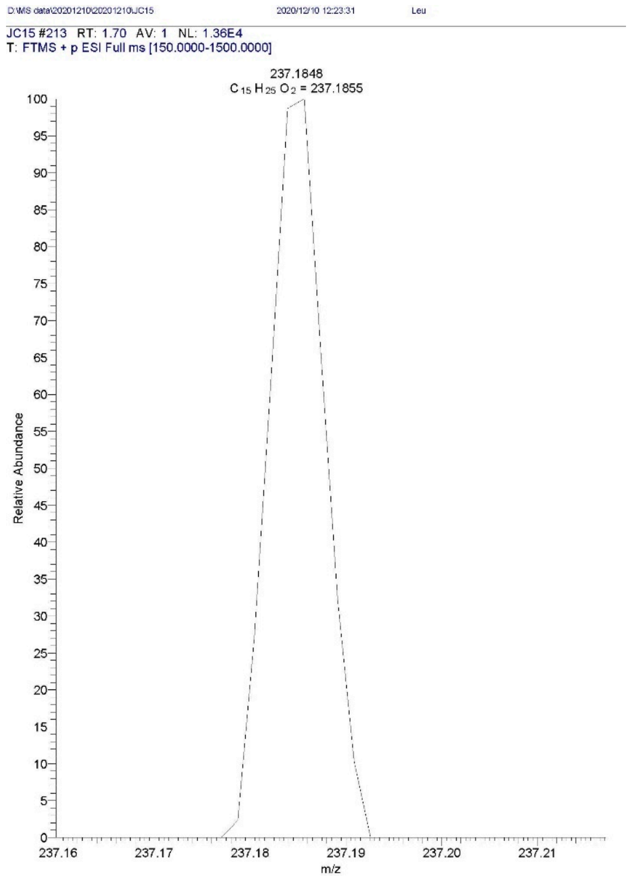

Figure S6. HRESIMS spectrum of cedrol-13-al (**2**)

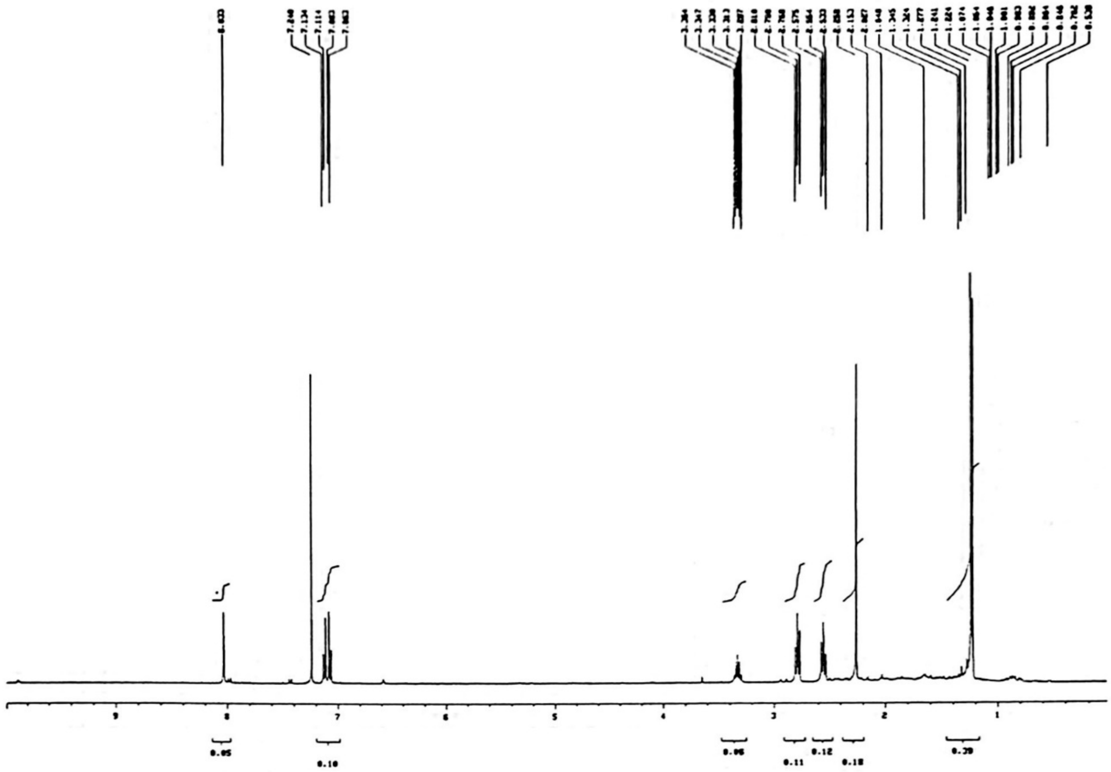

Figure S7. ^1H NMR spectrum of α-corocalen-15-oic acid (**3**) in $CDCl_3$ at 400 MHz

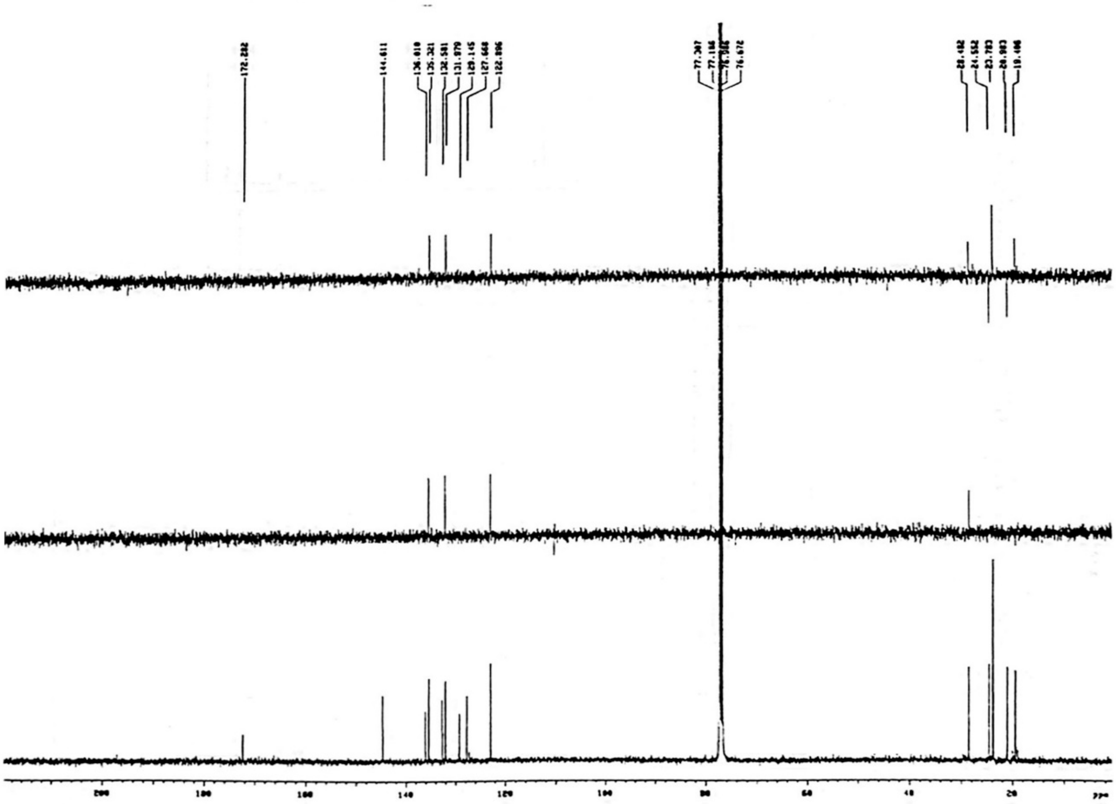

Figure S8. DEPT spectrum of α-corocalen-15-oic acid (**3**)

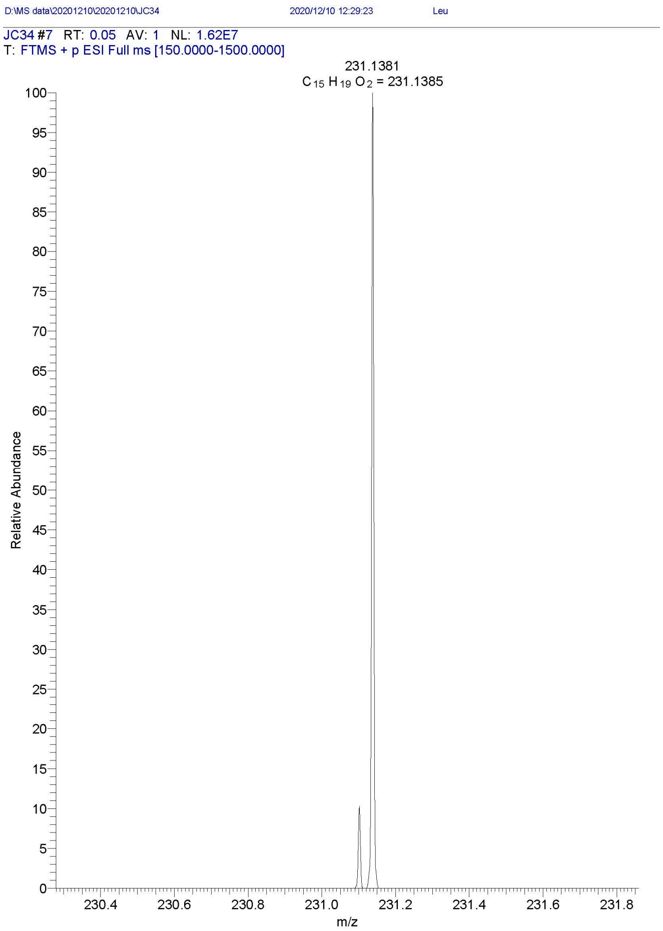

Figure S9. HRESIMS spectrum of α-corocalen-15-oic acid (**3**)

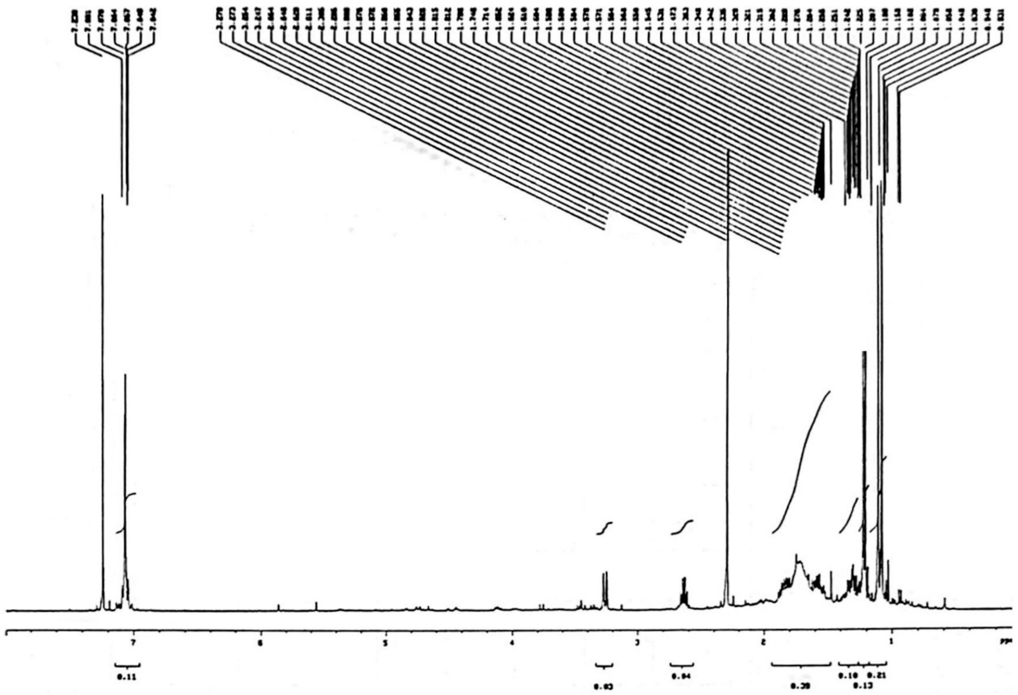

Figure S10. ^{1}H NMR spectrum of 1,3,5-bisaoltrien-10-hydroperoxy-11-ol (**4**) in $CDCl_3$ at 400 MHz

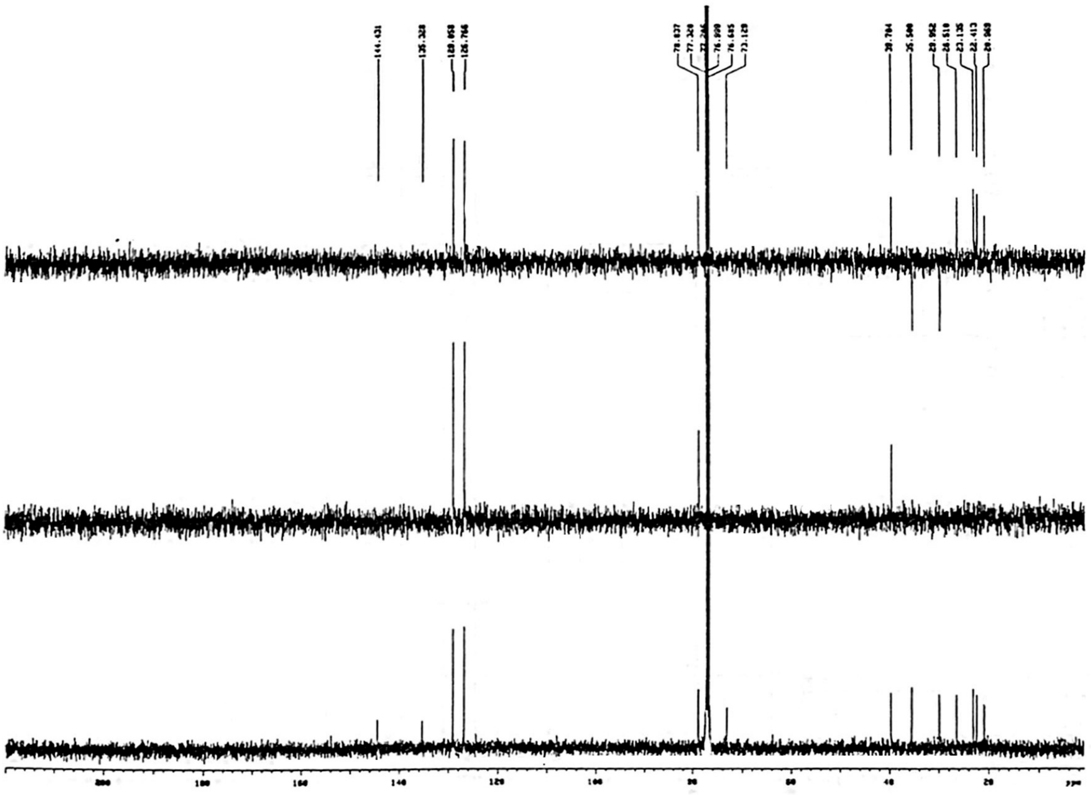

Figure S11. DEPT spectrum of 1,3,5-bisaoltrien-10-hydroperoxy-11-ol (**4**)

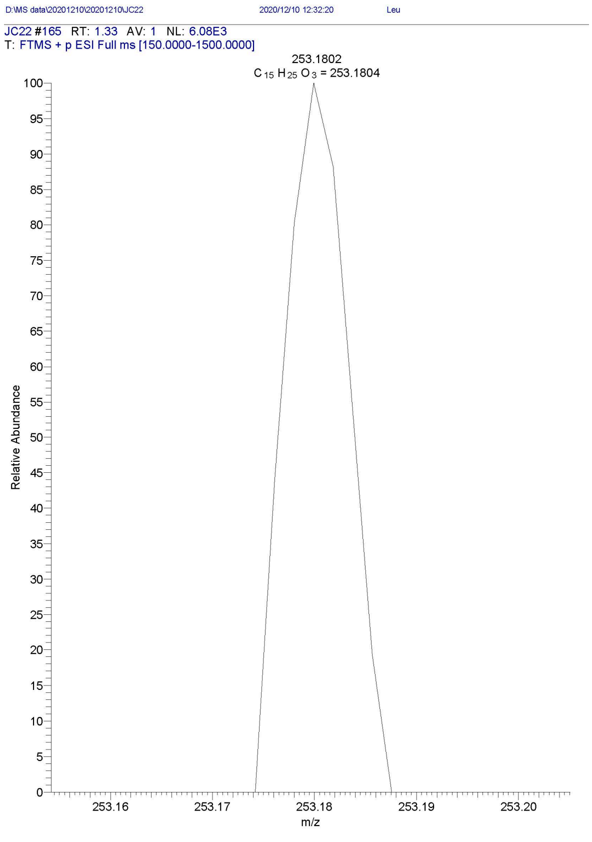

Figure S12. HRESIMS spectrum of 1,3,5-bisaoltrien-10-hydroperoxy-11-ol (**4**)

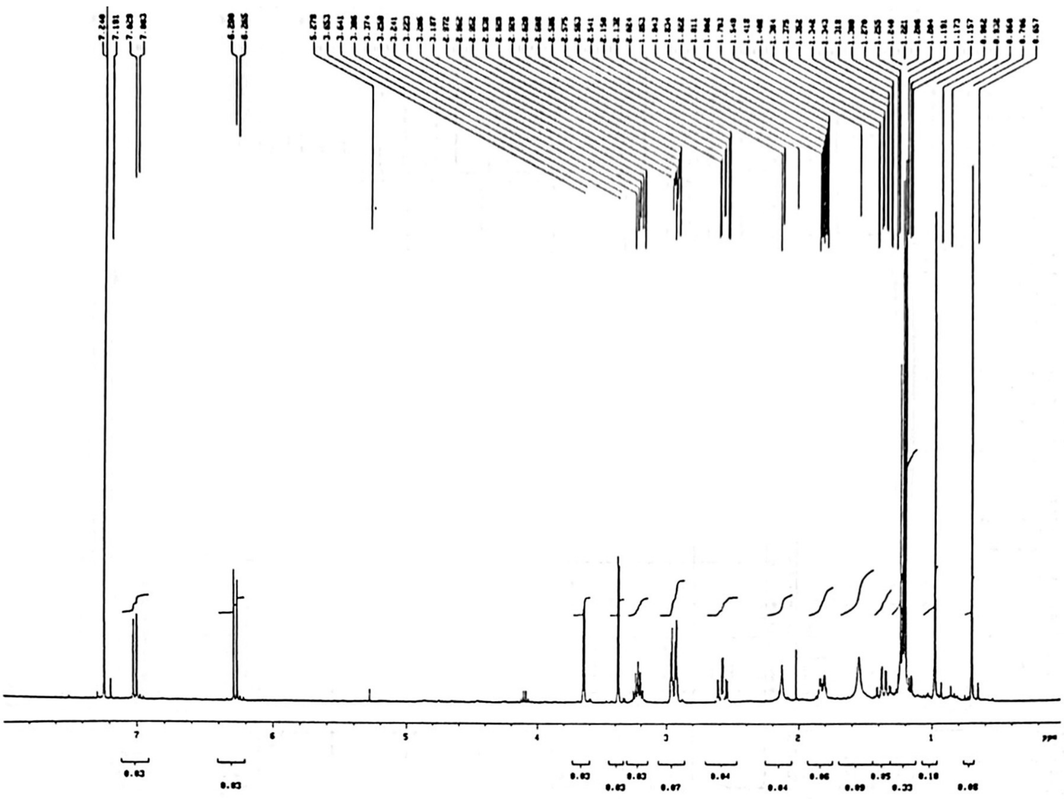

Figure S13. ^{1}H NMR spectrum of 1β,2β-epoxy-9α-hydroxy-8(14),11-totaradiene-3,13-dione (**5**) in $CDCl_3$ at 400 MHz

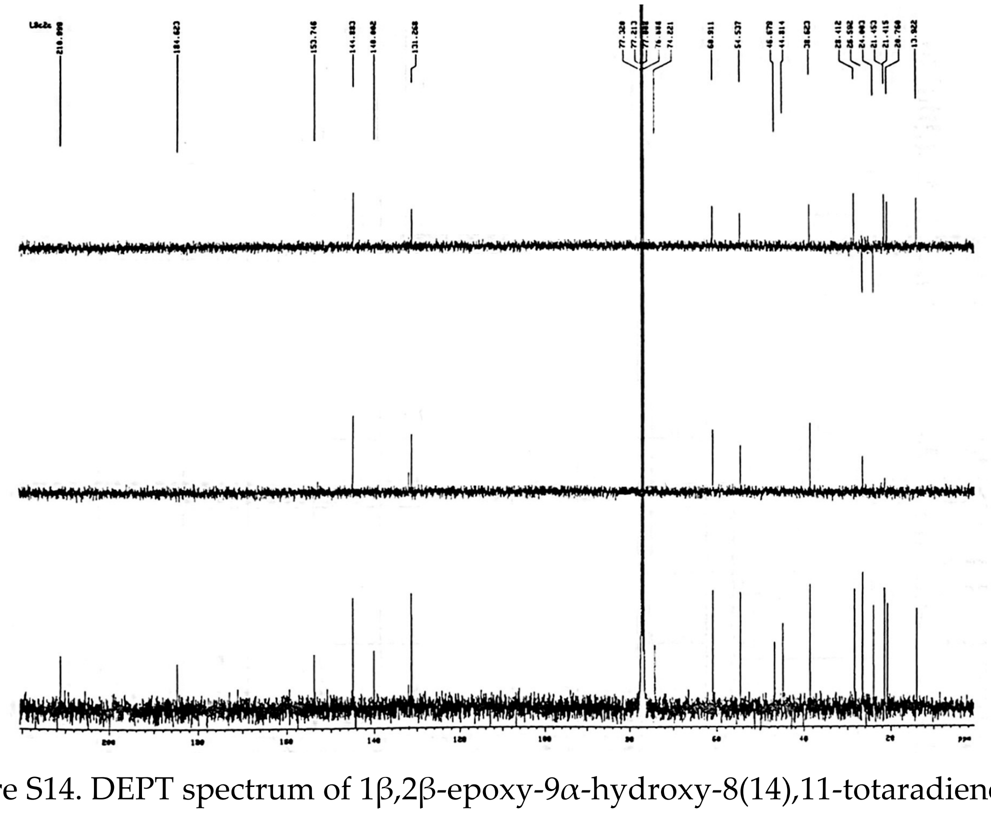

Figure S14. DEPT spectrum of 1β,2β-epoxy-9α-hydroxy-8(14),11-totaradiene-3,13-dione (**5**) in $CDCl_3$ at 400 MHz

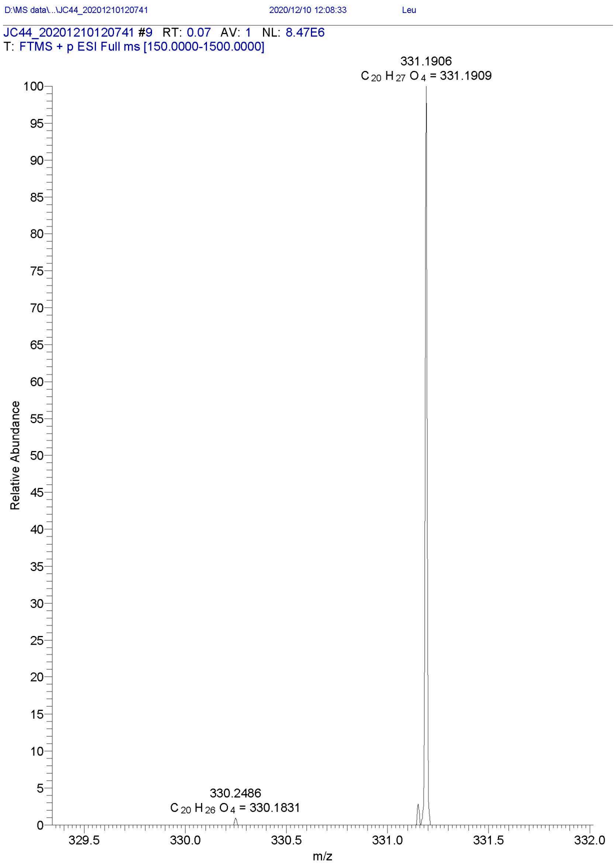

Figure S15. HRESIMS spectrum of 1β,2β-epoxy-9α-hydroxy-8(14),11-totaradiene-3,13-dione (**5**) in $CDCl_3$ at 400 MHz
